# Measurement invariance alignment of the burnout assessment tool (BAT-12) across 29 countries and six continents

**DOI:** 10.1038/s41598-026-50601-3

**Published:** 2026-04-28

**Authors:** Jacqueline Brassey, Leon T. De Beer, Roxy Merkand, Bradley A. Herbig, Wilmar B. Schaufeli, Hans De Witte, Kim Rubenstein

**Affiliations:** 1 School of Business and Economics, Vrije Universiteit Amsterdam, De Boelelaan 1105, Amsterdam, 1081 HV The Netherlands; 2 McKinsey Health Institute, Luxembourg, Luxembourg; 3 McKinsey Health Institute, Toronto, Canada; 4 McKinsey Health Institute, Washington, DC USA; 5 McKinsey Health Institute, Newark, New Jersey USA; 6 Department of Psychology, Norwegian University of Science and Technology (NTNU), Trondheim, Norway; 7 WorkWell Research Unit, Potchefstroom Campus, North-West University, Potchefstroom, South Africa; 8 Department of Psychology, Utrecht University, Utrecht, The Netherlands; 9 02L-WOPP- KU Leuven, Leuven, Belgium; 10 Optentia Research Unit, North-West University, Potchefstroom, South Africa

**Keywords:** Health care, Neuroscience, Psychology, Psychology

## Abstract

This study examines the cross-cultural measurement invariance of the 12-item burnout assessment tool (BAT-12) among 29,433 working adults from 29 countries across six continents. We employed a bifactor exploratory structural equation modeling specification with both fixed and free alignment methods. Across all parameters (loadings and thresholds), 92.62% were invariant. Across the countries, 91.09% of the item intercepts/thresholds and 95.69% of general-factor item loadings were invariant. The specific factor loadings were also highly invariant: mental distance 99.14%, exhaustion 89.94%, emotional impairment 92.53%, and cognitive impairment 91.95%, indicating evidence for the cross-national measurement invariance of the BAT-12. Furthermore, we also illustratively report the means for the cross-country comparisons which showed differences in latent global burnout scores according to this study’s samples. The evidence for validity shows that the BAT-12 reliably captures a global burnout dimension from the symptom-factor indicators of its four core symptoms, underscoring the BAT-12’s robust psychometric properties across diverse cultural contexts. Practically, the BAT-12 offers a more concise instrument for monitoring burnout complaints in multinational settings, guiding managers, occupational health professionals, and researchers in developing and evaluating burnout prevention and intervention strategies.

## Introduction

Work-related stress has been increasing throughout recent years. Burnout, a syndrome of inability and withdrawal affects employees in all professional contexts, exacting a heavy toll on them, their organisations, and healthcare systems across the globe^1,2^. Unsurprisingly, throughout the last half-century, burnout has become a hot topic and a major concern in modern industrialised and post-industrial societies. The spread of burnout across numerous professional sectors (e.g. healthcare, education, community, business, and military) in recent decades has stimulated intense discussion, research, and debate regarding its aetiology, phenomenology, and treatment, leading to a growing array of studies and articles on the topic.As organizations increasingly operate across borders and researchers conduct multinational studies, the need to accurately assess burnout across diverse cultural contexts has become critical^3^–^5^. However, without demonstration that burnout instruments are measurement invariant across countries, observed differences in burnout scores may reflect artifacts of translation, differential item functioning, or cultural response styles rather than true differences in the underlying syndrome. This measurement challenge is particularly important for burnout, which must be distinguished from broader mental health or wellbeing constructs through theoretically grounded, psychometrically sound assessment tools.

Despite extensive research attention, significant debates continue regarding burnout’s conceptualization, including its distinctiveness from depression and general psychological distress, its role in the job demands-resources (JD-R) model, and whether it constitutes a coherent syndrome or represents work-specific manifestations of broader mental health conditions^6–8^. These theoretical discussions remain important for advancing the field All these debates emphasised the need for improved and more theoretically founded approaches to burnout assessment, given its complex and multifaceted nature. Indeed, burnout is an important and pragmatic concept for occupational health experts within organisations to address employee strain^1^.

In response to these issues, the Burnout Assessment Tool (BAT) was developed as a novel instrument to address the limitations of previous measures of burnout and thereby updating the conceptualisation and measurement of burnout syndrome^9^. BAT-defined burnout is “a work-related state of exhaustion that occurs among employees, characterised by extreme tiredness, reduced ability to regulate cognitive and emotional processes, and mental distancing”^9^, p. 4]. The BAT’s conceptual framework (based on a theoretical synthesis of contemporary burnout research and interviews with occupational health professionals who work with burnout) is multidimensional, comprising four core symptoms of exhaustion (fatigue and extreme tiredness), mental distance (withdrawal and cynicism), and cognitive and emotional impairment (loss of regulation over cognitive and emotional processes, manifesting as, for example, memory lapses or emotional outbursts); providing a more complete representation of the burnout syndrome^9^. The 23-item BAT measure also exhibits excellent psychometric properties and has been tested and validated across different occupational and cultural contexts^10–12^. This provides researchers and practitioners with an alternative to traditional burnout measures.The BAT was developed as an alternative approach that enables researchers to model burnout as a multidimensional syndrome with a global factor, a theoretical position that contrasts with the MBI manual’s caution against computing total burnout scores^13,14^. This difference reflects ongoing theoretical debates about whether burnout should be conceptualized as a unified syndrome or as separate, related dimensions which is which is central to the conceptualization of burnout as a syndrome and for which the BAT has provided empirical support^10–12^.

In the present study, “cross-cultural” is used in a comparative measurement sense to refer to BAT-12 comparability across country samples from different national and linguistic contexts, rather than to culture as a directly measured variable. For this reason, the study focuses on whether the BAT-12 demonstrates sufficient measurement invariance across countries to support meaningful comparisons of latent burnout scores. Although the original 23-item BAT has been validated across multiple occupational and cultural contexts^10–12^, no large cross-cultural study has yet examined the shortened BAT-12 across a broad range of countries^15^. Prior work suggested that reducing the BAT-23 to 12 items involved only limited information loss^15^, which makes the BAT-12 a promising option where assessment economy is important. However, item reduction is not psychometrically neutral, particularly in multinational research. Shorter forms may reduce content coverage and indicator redundancy, and may be more sensitive to item-level non-invariance if some items function differently across groups. While measurement invariance has been established for the BAT-23 and BAT-12 in specific national settings, including Norwegian and South African samples^16,17^, this does not by itself establish comparability across a substantially broader set of countries. For this reason, the cross-cultural applicability of the BAT-12 requires direct empirical evaluation across countries.

Without studies that involve large, multinational samples, it is difficult to assess whether burnout, as measured by the BAT-12, is truly cross-culturally applicable and whether it provides equivalent assessments of burnout scores across different groups. Furthermore, shortened versions of instruments can be essential for practitioners who want to measure burnout levels in organization where time constraints away from the job are a concern. Therefore, establishing measurement invariance is important in cross-cultural research because it ensures that observed differences between groups are due to genuine differences in the construct and not to artefacts of measurement or cultural bias.

For example, the bifactor exploratory structural equation modelling (BESEM) representation of the BAT has been shown to be a superior representation; comprising a global burnout score while also including the four component factors^11,12^. This representation allows for the calculation of a global burnout score, while retaining the importance of the multidimensional nature of the syndrome in calculating that global score. Therefore, bifactor-ESEM shows stronger evidence compared to traditional confirmatory factor analysis and allows for more nuanced and flexible modelling of complex psychological constructs such as burnout which is considered a syndrome. Nevertheless, it should be noted that the second-order representation of the BAT has also shown promise^10^, but that there has been criticism surrounding the use of second-order models when the interest is an accurate global score^18^.

Through a comprehensive investigation of the BAT-12 in 29 diverse countries across six continents, this study addresses the gap in the existing literature regarding the cross-cultural use of the BAT-12 while also presenting levels of burnout across the 29 countries. More specifically, the present study uses the factor alignment approach within the bifactor-ESEM framework to explore the underlying cross-cultural equivalence of the BAT-12 factor structure and item parameters across 29 country samples. The factor alignment approach allows for a more nuanced exploration of the invariance of the BAT-12 measurement model, combining the flexibility of ESEM with the parsimony of bifactor models. The alignment method is especially useful for the assessment of measurement invariance in large-scale, multigroup studies because it can handle many groups and is more realistic than the traditional multigroup CFA^19,20^.

This study aims to examine the validity of comparing burnout means using the BAT-12 across diverse cultural contexts in both the global north and south. Given burnout’s significance as a major occupational health issue, establishing measurement invariance for the BAT-12 across multiple countries could potentially advance our understanding of burnout’s cross-cultural manifestation and inform international research and practice in this area.

## Method

### Participants and procedure

**Fig. 1:**
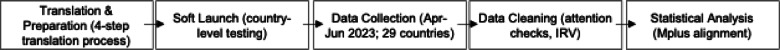
Study design and chronology.

We partnered with several online panel platforms (Dynata, Clint, and Borderless Access) to recruit working adults across 29 countries. These platforms were also used to program and administer the survey (see Table 1 for demographics by country) (More details can be obtained by the first author upon request). The collection of data on the Burnout Assessment Tool was part of a larger research project on employee health by McKinsey Health Institute. Data was collected between April and June 2023 and all respondents received a financial incentive for providing their time to participate in the survey. The study followed a sequential process including translation and survey preparation, pilot testing (soft launch), full data collection (April–June 2023), and data cleaning and analysis (see Fig. 1).

Across crowdsourcing platforms, we aimed to collect approximately 1000 participants per country to ensure a conservative 90% power for detecting small between-country effects (Cohen’s d = 0.15) while allowing for data exclusions due to missingness or careless responders in each country. Power calculations showed that d = 0.15 requires ~ 935 respondents per country to detect small between-country effects. Therefore, we conservatively aimed to collect ~ 1000 respondents per country.

This study was carried out in accordance with relevant guidelines and regulations, including the Declaration of Helsinki and applicable data protection regulations. This study did not undergo review by an Institutional Review Board (IRB), as it was conducted within a corporate setting that does not operate under an IRB framework. Instead, the research protocol was reviewed and approved by the McKinsey Health Institute Approval Board, which includes an internal Risk and Compliance Team, which evaluated the study’s ethical implications, data handling practices, and compliance with applicable privacy and legal standards per geographic region (e.g., GDPR, HIPAA). All data used in this study were fully anonymized by the panel providers prior to transfer to the research team, ensuring cross-border data transfers occurred under appropriate legal safeguards. All participants provided informed consent prior to participating in the survey, including consent for data collection, processing, and use for research purposes. Participation was voluntary, and participants were informed they could withdraw at any time without penalty. All data were collected and stored in compliance with applicable privacy and data protection standards.

From the sample collected, we removed participants who failed one or more of our eight attention check questions where they were asked to select ‘Strongly Disagree’ as their response. Additionally, we removed respondents with outlier values on intra-individual response variability (IRV)^21^. After these removals, the final sample size was 29,433 respondents available for further analyses. Overall, the mean age of the sample was 39.45 (SD = 10.63) years with slight majority of the sample indicating female as their gender (49.28%) and a majority of the overall sample had a tertiary education (62.10%). See Table 1 for more information and a breakdown per country.

The English version of the BAT-12 was translated by an expert translation-services company into 17 languages (Arabic, Simplified Chinese, Dutch, French (Canada), French (Europe), German, Hindi, Indonesian, Italian, Japanese, Korean, Polish, Portuguese, Spanish, Swedish, Turkish, and Thai) to ensure equivalence of item presentation to the participants. More, specifically, the scale translation was conducted in four steps. In step 1, the BAT-12 items were provided to an external vendor who conducted a forward-translation of the items into the target language(s). This vendor maintains a network of native-speaking freelance translators who reside in their native language countries and are reimbursed for their translation services. Each translation was reviewed by the vendor for coherence, terminological consistency, and language quality. In step 2, we asked native-language editors to back-translate the output of step 1 into English and edit translations for coherence. In step 3, we examined if there were any discrepancies between the provided back-translation and the original English items. Pre-final items in English and the target language were provided in writing to the editors from step 2 for their review. In this step, editors focused on translation for meaning equivalence rather than literal translation. In the fourth and final step, we reviewed the final items with survey vendors in each country ahead of soft launching our survey in each target country to ensure items would be understood.

**Table 1:** Participant demographics by Country.

Country	Count (*n*)*	Mean age (SD)	Gender (% female)	Education (% Tertiary)	Position (% Manager)
AE	1065	38.6 (8.49)	33%	69%	26%
AR	1085	39.2 (9.90)	51%	62%	9%
AU	1037	40.2 (11.67)	58%	71%	26%
BR	1087	38.3 (12.20)	48%	50%	21%
CA	1020	43.4 (11.92)	56%	69%	20%
CH	823	38.6 (11.80)	36%	93%	22%
CL	975	37.3 (10.28)	58%	53%	7%
CM	303	31.4 (7.17)	35%	90%	15%
CN	1002	37.7 (10.32)	44%	62%	43%
CO	1087	36.3 (10.07)	43%	64%	6%
DE	1040	46.0 (12.07)	54%	58%	12%
EG	1028	36.1 (9.59)	48%	71%	42%
FR	1045	44.7 (10.86)	61%	51%	14%
ID	1072	32.9 (9.00)	37%	47%	24%
IT	1041	44.2 (11.46)	49%	30%	13%
JP	1016	47.6 (9.62)	41%	56%	11%
KR	1000	42.3 (9.70)	48%	77%	17%
MX	1078	36.9 (9.98)	47%	62%	16%
NG	1225	33.1 (9.83)	46%	72%	30%
NL	1035	45.8 (12.21)	58%	65%	17%
NZ	1035	37.2 (11.63)	66%	57%	19%
PL	1037	38.3 (11.07)	54%	69%	13%
SA	988	34.9 (9.47)	39%	53%	24%
SE	1050	40.7 (12.38)	52%	42%	15%
SG	970	40.2 (10.70)	43%	76%	36%
TR	993	38.5 (9.87)	48%	49%	25%
UK	1000	44.1 (12.48)	56%	59%	22%
US	1184	46.5 (13.39)	58%	71%	26%
ZA	1091	33.4 (9.27)	62%	53%	25%
OVERALL	29 412*	39.5 (10.63)	49%	62%	21%

AE = United Arab Emirates, AR = Argentina, AU = Australia, BR = Brazil, CA = Canada, CH = Switzerland, CL = Chile, CM = Cameroon, CN = China, CO = Colombia, DE = Germany, EG = Egypt, FR = France, ID = Indonesia, IT = Italy, JP = Japan, KR = South Korea, MX = Mexico, NG = Nigeria, NL = Netherlands, NZ = New Zealand, PL = Poland, SA = Saudi Arabia, SE = Sweden, SG = Singapore, TR = Türkiye, UK = United Kingdom, US = United States, ZA = South Africa; * = values obtained from the multigroup analyses in Mplus based on included data in the analysis.

### Measure

Burnout complaints was measured with the Burnout Assessment Tool 12-item version (BAT-12)^15^. The BAT-12 measures four component-symptoms of burnout: Exhaustion (3 items), e.g. “At work, I feel mentally exhausted”; Mental Distance (3 items), e.g. “I’m cynical about what my job means to others”; Cognitive Impairment (3 items), e.g., “When I’m working, I have trouble concentrating”; Emotional Impairment (3 items), e.g., “At work, I may overreact unintentionally”. The broader survey measured several constructs related to demands (e.g., work pressure) and resources (e.g., career opportunities, supervisor support) in the workplace. The survey also measured several health-related outcomes (e.g., distress symptoms), and work-related outcomes (e.g., intent to leave). Respondents first rated their health outcomes, then their work outcomes, then randomized blocks of questions about demands and resources. At the end of the survey, respondents rated their demographic characteristics (e.g.,, income, age, gender). Block randomization was applied within and between countries, and items within blocks were randomized. Each item within the survey was considered voluntary, and respondents were able to skip any items in the survey they did not wish to respond to, including the BAT-12 items. If a respondent did not provide a response to an item within the survey, they were provided with a reminder to complete that question before moving to the next section of the survey. No missing data was imputed, but with our chosen estimation method results are based on pairwise present data. Therefore, cases were not completely excluded if they had missing data and all present information was used in the estimation process.

### Data analysis

We used Mplus 8.11^22^ to analyse the data. Specifically, due to the large number of countries in our data, we used the multi-group alignment procedure with the expected bifactor exploratory structural equation modelling solution found in other cross-country studies^12^. See Fig. 1 below for a conceptual illustration of the model. We applied an orthogonal target rotation because orthogonality between the general and specific factors is consistent with the intended bifactor decomposition, in which the general factor captures the variance common to all items and the specific factors capture residual domain-specific variance after accounting for the general factor^23^. This specification also aligns with prior BAT bifactor research^11,12^. As with any bifactor model, the substantive interpretability of the specific factors depends on the extent of reliable residual variance remaining once the general factor has been extracted. Although ESEM is considered exploratory, employing a target rotation enables a confirmatory approach by allowing the expected target loadings to be specified as such for specific factors while allowing for approximately-zero cross-loadings^23,24^. Due to the categorical nature of the data, we used the mean- and variance-adjusted weighted least squares estimator (WLSMV). We calculated McDonald’s omega coefficients as estimates of reliability^23,25^. Furthermore, we did not correlate any between-item errors. No missing data was imputed, but with our chosen estimation method results are based on pairwise present data.

**Fig. 2:**
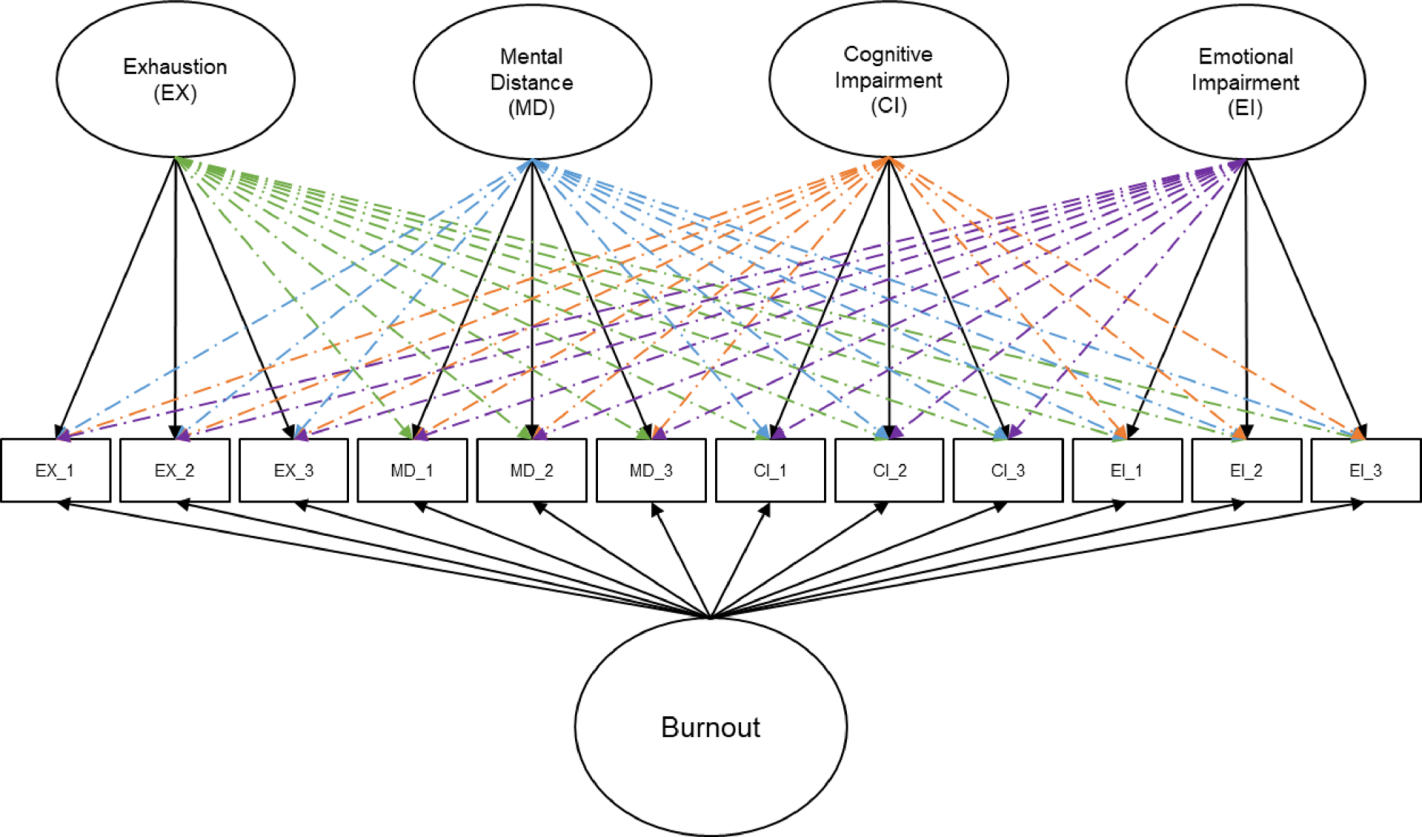
The conceptual bifactor-ESEM model implemented in the alignment procedure.

Model fit was assessed using the Root Mean Square Error of Approximation (RMSEA), the Comparative Fit Index (CFI), the Tucker-Lewis Index (TLI), and the Standardized Root Mean Squared Residual (SRMR). The alignment method is designed to estimate an approximately invariant model across many groups, even in the presence of potential minor variations^19,20,26,27^. The procedure allows for the comparison of latent factor means across groups by aligning the model parameters to minimize non-invariance. The aim was to model global burnout scores, indicated by the items of four component factors (see Fig. 2 below for a conceptual illustration), for which equivalent means could be presented. For completeness, we tested both the free and fixed alignment methods for latent burnout scores, as well as the observed mean scores.

## Results

### Model fit statistics per country and overall

In line with good practice, we modelled the BESEM structure in each country to ascertain if the model fit. We also tested an overall model with the pooled sample. In all instances the BESEM model was an excellent fit to the data (see Table 2 below). The calculated McDonald’s omega coefficients, as indicator of reliability, showed that the overall global burnout score was also highly acceptable in all cases, ranging from ω = 0.934 to 0.982.

### Measurement invariance alignment

Our initial analyses indicated that free alignment was not the most suited solution to the data. Specifically, Mplus showed a warning that the standard error comparison indicated that the free alignment model may be poorly identified and that using the fixed alignment option may resolve this problem. Therefore, we deferred to the fixed alignment method with the Netherlands as the reference group, which resulted in no warning. The Netherlands was chosen as this country officially considers burnout an occupational disease classification and therefore makes sense as the logical comparison group. The Netherlands is also one of the homes to the language (Dutch) in which the original BAT was tested in both the Netherlands and Belgium^9^.

The alignment analyses showed excellent model fit (χ2 = 1238.027; df = 464; *p* < .001; CFI = 0.999; TLI = 0.995; SRMR = 0.006; RMSEA = 0.041 [0.038, 0.043]).Specifically, the analysis showed that 92.62% (2,901) of the 3,132 parameters assessed were invariant, indicating that the BAT-12 captures burnout consistently across countries with limited evidence of significant bias across groups. Importantly, none of the parameter groups approached the commonly cited 25% or the more recently suggested 20% ESEM rule-of-thumb thresholds for non-invariance^27^. At the same time, these thresholds should be interpreted as heuristic guidelines rather than definitive proof that all latent mean comparisons are unaffected by the remaining non-invariance.

A detailed examination of the parameters further highlighted the instrument’s reliability. Among the intercepts and thresholds, 91.45% (1,273 of 1,392) were found to be invariant, demonstrating that participants across countries interpret and respond to the items in a similar manner. The factor loadings, which reflect how well each item measures its intended construct, also exhibited high levels of invariance across the BAT-12’s core dimensions.

For the general burnout factor, 95.69% (333 of 348) of the loadings were invariant, indicating that the overarching construct of burnout is captured consistently across all groups. Among the specific dimensions of the BAT-12, mental distance showed the highest invariance (99.14%, or 345 of 348 parameters), followed by emotional impairment (92.53%, 322 of 348), cognitive impairment (91.95%, 320 of 348) and exhaustion (89.94%, 313 of 348). These results highlight the BAT-12’s capacity to measure both general and specific aspects of burnout with remarkable consistency, even across culturally diverse samples. Note that due to the length of all the alignment output, which would be impractical to add to the manuscript, it is provided in the online supplementary material at: https://doi.org/10.17605/OSF.IO/73VD8.

Overall, these findings affirm evidence for the validity of the BAT-12 as tool for assessing burnout complaints in international research. The high alignment of parameters across countries not only supports the validity of the instrument but also ensures that cross-group comparisons of burnout levels and profiles are meaningful and unbiased. Notably, the general burnout factor underlying the four component factors exhibited exceptional cross-cultural stability. While dimensions such as emotional impairment and exhaustion showed more instances of non-invariance, their results remain within acceptable ranges and well below any rule of thumb thresholds that might signal concern about invariance. These findings provide support for the BAT-12’s applicability in cross-cultural research, meeting and exceeding standards for approximate measurement equivalence^27^. Therefore, we continued with presenting the levels of burnout across the countries.

**Table 2:** The BESEM fit statistics per individual country and for the pooled sample.

	Country	χ^2^	df	RMSEA [90% CI]	CFI	TLI	SRMR	ω
1.	Argentina	32.225	16	0.031 [0.015, 0.046]	0.999	0.995	0.006	0.954
2.	Australia	28.053	16	0.027 [0.008, 0.043]	0.999	0.997	0.005	0.958
3.	Brazil	24.786	16	0.022 [0.001, 0.039]	0.999	0.998	0.006	0.934
4.	Cameroon	19.097	16	0.025 [0.001, 0.061]	0.999	0.997	0.010	0.947
5.	Canada	30.214	16	0.030 [0.012, 0.045]	0.999	0.997	0.005	0.963
6.	Chile	28.381	16	0.028 [0.009, 0.045]	1.000	0.999	0.005	0.972
7.	China	32.753	16	0.032 [0.016, 0.048]	1.000	0.998	0.005	0.976
8.	Colombia	23.777	16	0.021 [0.001, 0.038]	1.000	0.998	0.005	0.964
9.	Egypt	52.770	16	0.047 [0.033, 0.062]	0.999	0.994	0.006	0.967
10.	France	28.774	16	0.028 [0.010, 0.044]	0.999	0.997	0.006	0.954
11.	Germany	23.661	16	0.021 [0.001, 0.039]	1.000	0.999	0.004	0.965
12.	Indonesia	34.438	16	0.033 [0.018, 0.048]	0.999	0.997	0.005	0.968
13.	Italy	53.445	16	0.047 [0.034, 0.062]	0.998	0.992	0.008	0.958
14.	Japan	28.807	16	0.028 [0.010, 0.044]	1.000	0.998	0.004	0.964
15.	Mexico	48.749	16	0.044 [0.030, 0.058]	0.998	0.994	0.007	0.966
16.	Netherlands	58.229	16	0.050 [0.037, 0.065]	0.998	0.991	0.007	0.959
17.	Nigeria	33.936	16	0.030 [0.016, 0.044]	0.999	0.996	0.006	0.949
18.	Poland	42.418	16	0.040 [0.026, 0.055]	0.999	0.996	0.006	0.970
19.	Saudi Arabia	17.920	16	0.011 [0.001, 0.032]	1.000	1.000	0.003	0.972
20.	Singapore	22.016	16	0.020 [0.001, 0.038]	1.000	0.999	0.004	0.964
21.	South Africa	20.025	16	0.015 [0.001, 0.034]	1.000	0.999	0.005	0.936
22.	South Korea	76.717	16	0.062 [0.048, 0.076]	0.997	0.987	0.009	0.960
23.	Sweden	49.145	16	0.044 [0.031, 0.059]	0.998	0.993	0.007	0.967
24.	Switzerland	27.951	16	0.030 [0.009, 0.048]	0.999	0.997	0.006	0.962
25.	Turkey	44.552	16	0.042 [0.028, 0.057]	0.999	0.997	0.004	0.982
26.	United Arab Emirates	41.624	16	0.039 [0.025, 0.053]	0.999	0.996	0.006	0.964
27.	United Kingdom	67.867	16	0.057 [0.043, 0.071]	0.997	0.987	0.008	0.959
28.	United States	39.682	16	0.035 [0.022, 0.049]	0.999	0.996	0.005	0.966
29.	New Zealand	19.837	16	0.015 [0.001, 0.034]	1.000	0.999	0.005	0.953
=	Pooled sample	461.549	16	0.030 [0.028, 0.033]	0.999	0.997	0.003	0.963

*χ*^2^: chi-square; df: degrees of freedom; RMSEA: Root Mean Square Error of Approximation; CFI: Comparative Fit Index; TLI: Tucker-Lewis Index; SRMR: Standardized Root Mean Squared Residual; ω: McDonald’s omega coefficient as indicator of construct reliability.

### Latent burnout scores across countries

Table 3 presents the relative ordering of country-specific latent means generated by the alignment model. However, these comparisons should be interpreted descriptively and with caution, particularly because many countries in the middle of the distribution are closely clustered and because the samples were not nationally representative.

**Table 3:** Latent burnout scores across the countries based on the fixed alignment analysis.

Rank*	Country		Groups with significantly lower mean burnout level (*p* < .05)
1	SG	0.450	AE, SE, CL, AU, KR, CH, NG, ID, PL, CN, DE, FR, IT, US, CM, AR, MX, TR, CO
2	SA	0.361	CL, AU, KR, CH, NG, ID, PL, CN, DE, FR, IT, US, CM, AR, MX, TR, CO
3	EG	0.344	CL, AU, KR, CH, NG, ID, PL, CN, DE, FR, IT, US, CM, AR, MX, TR, CO
4	NZ	0.279	CL, AU, KR, NG, ID, PL, CN, DE, FR, IT, US, CM, AR, MX, TR, CO
5	TR	0.270	PL, FR, US, CM, AR, MX, TR, CO
6	CA	0.208	NG, ID, PL, DE, FR, IT, US, CM, AR, MX, TR, CO
7	GB	0.134	CN, DE, IT, US, CM, AR, MX, TR, CO
8	SE	0.109	PL, DE, FR, IT, US, CM, AR, MX, TR, CO
9	CL	0.106	PL, FR, US, CM, AR, MX, TR, CO
10	JP	0.104	CN, DE, FR, IT, US, CM, AR, MX, TR, CO
11	AU	0.048	FR, US, CM, AR, MX, TR, CO
12	ZA	0.018	US, CM, AR, MX, TR, CO
13	KR	0.006	CM, AR, MX, TR, CO
14	**NL**	**0.000**	IT, US, CM, AR, MX, TR, CO
15	NG	-0.002	US, CM, AR, MX, TR, CO
16	AE	-0.027	IT, US, CM, AR, MX, TR, CO
17	ID	-0.042	AR, MX, TR, CO
18	PL	-0.117	TR, CO
19	CN	-0.129	AR, MX, TR, CO
20	DE	-0.145	AR, MX, TR, CO
21	FR	-0.156	AR, MX, TR, CO
22	BR	-0.182	
23	IT	-0.249	MX, CO
24	US	-0.261	MX, CO
25	CM	-0.288	
26	AR	-0.428	
27	MX	-0.430	
28	CH	-0.454	
29	CO	-0.482	

* = Rank based on descending factor intercepts from alignment analysis; SG = Singapore, SA = Saudi Arabia, EG = Egypt, NZ = New Zealand, AE = United Arab Emirates, CA = Canada, SE = Sweden, JP = Japan, CL = Chile, UK = United Kingdom, AU = Australia, KR = South Korea, NL = Netherlands, NG = Nigeria, ID = Indonesia, CH = Switzerland, ZA = South Africa, PL = Poland, CN = China, DE = Germany, FR = France, BR = Brazil, US = United States, IT = Italy, CM = Cameroon, AR = Argentina, MX = Mexico, TR = Turkey, CO = Colombia. NL is also the reference group.

In terms of relative comparisons, it should be noted that samples were not representative and results should be interpreted with caution. As can be seen from Table 3, Singapore (SG) exhibited the highest latent score for burnout among the countries, followed closely by Saudi Arabia (SA) and Egypt (EG). This is consistent with Fig. 2, where these countries are plotted toward the top of the distribution, reflecting the least negative latent intercepts and thus the highest burnout levels. The results also show which countries scored significantly lower than each respective country in terms of latent burnout, based on difference testing (*p* < .05). Importantly, this ranking must be interpreted relative to the reference group (the Netherlands, NL): higher intercepts correspond to higher latent burnout levels, while lower (more negative) intercepts indicate lower levels. Countries such as Colombia (CO), Mexico (MX), and Argentina (AR) are positioned at the bottom of the ranking, reflecting the lowest burnout scores. In the middle of the distribution lie countries such as South Korea (KR) and Japan (JP), which indicate more moderate burnout levels in this dataset. By contrast, Switzerland (CH) is positioned near the lower end of the ranking.

Table 3 further highlights that a large number of countries show significantly lower burnout compared to the top scorers. For example, Turkey (TR) scores significantly lower than many of the countries ranked above it, although several countries also score significantly lower than Turkey itself. Similarly, countries like Colombia (CO), Mexico (MX), and Argentina (AR) fall toward the lower end of the burnout ranking.

Although latent mean differences were observed across the country samples, these patterns should be treated as descriptive features of the present dataset rather than evidence of robust regional or national differences in population burnout levels. For example, in Asia, Singapore and Saudi Arabia report some of the highest burnout levels, whereas Japan and South Korea appear closer to the center of the ranking. Africa shows mixed results, with Egypt ranking higher than South Africa (ZA), while Cameroon (CM) and Nigeria (NG) sit in the mid-range. Among European countries, Sweden (SE) and the United Kingdom (UK) are higher, while Poland (PL) and the Netherlands (NL) are positioned among the lower scorers. In the Americas, Canada (CA) ranks relatively high, the United States (US) is mid-range, and Mexico (MX), Colombia (CO), and Argentina (AR) show relatively low burnout. In Oceania, New Zealand (NZ) scores somewhat higher than Australia (AU), suggesting elevated burnout levels in comparison.

### Comparative burnout scores by method

**Fig. 3:**
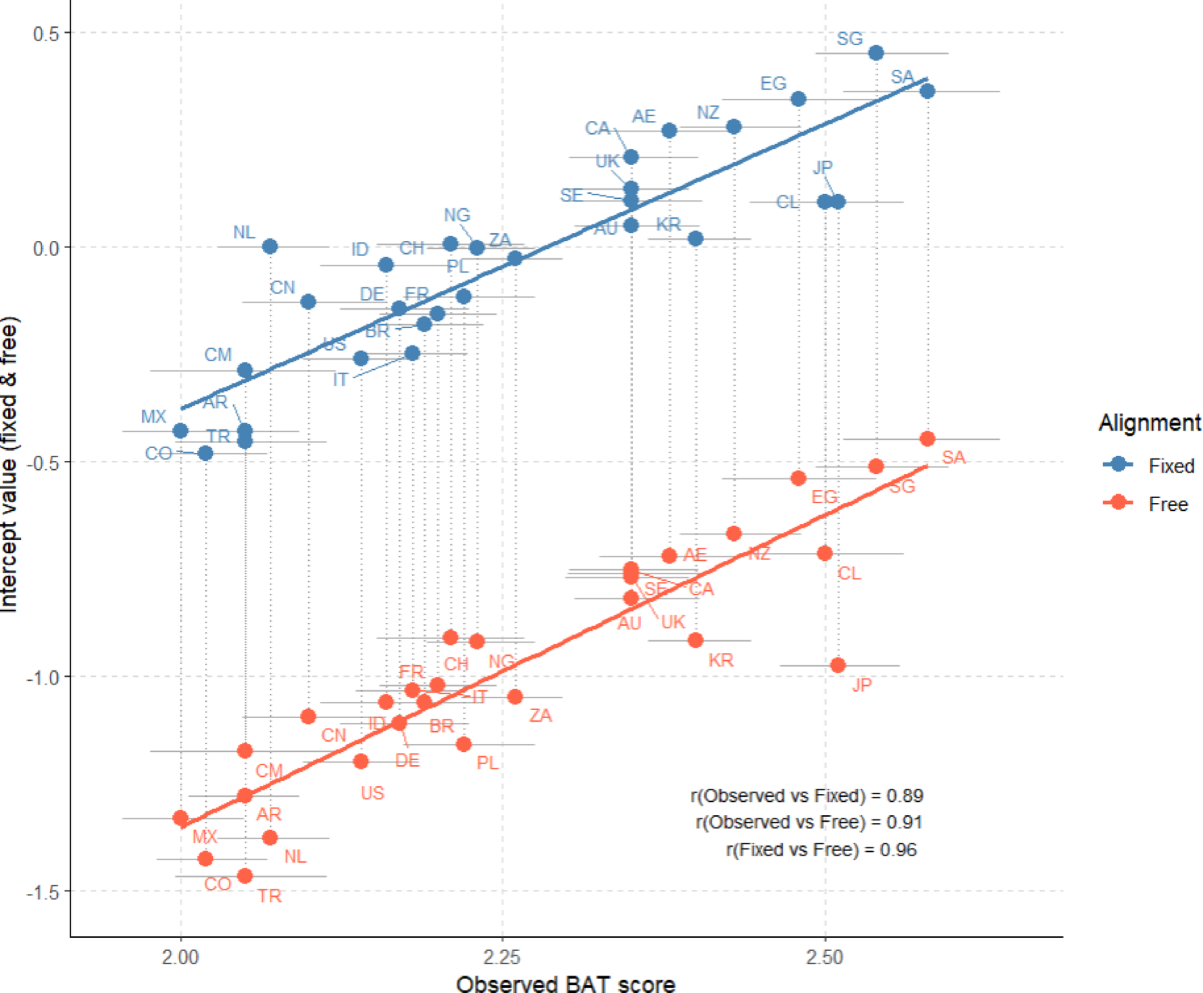
Fixed alignment, free alignment, and observed mean scores with 95% confidence intervals. SA = Saudi Arabia, SG = Singapore, EG = Egypt, NZ = New Zealand, AE = United Arab Emirates, CA = Canada, SE = Sweden, JP = Japan, CL = Chile, UK = United Kingdom, AU = Australia, KR = South Korea, NL = Netherlands, NG = Nigeria, ID = Indonesia, CH = Switzerland, ZA = South Africa, PL = Poland, CN = China, DE = Germany, FR = France, BR = Brazil, US = United States, IT = Italy, CM = Cameroon, AR = Argentina, MX = Mexico, TR = Turkey, CO = Colombia.

Figure 3 below plots all BAT scores for both the free alignment and fixed alignment methods compared to each other and also the observed mean score. Furthermore, presented on the figure is the correlation between scores which were all strongly correlated with each other (*r*s = 0.89-0.96). This indicates that countries with higher observed BAT scores also have higher model estimated intercepts, and vice versa, as would be expected.

Our results show that country latent means were highly consistent across fixed and free alignment solutions (*r* = .96). The highest observed BAT means in this sample were for Saudi Arabia (SA; 2.58), Singapore (SG; 2.54), Japan (JP; 2.51), Chile (CL; 2.50), and Egypt (EG; 2.48); these same countries were found at the high end of the latent intercept distributions under both fixed and free alignment. At the low end, Mexico (MX; 2.00), Colombia (CO; 2.02), Turkey (TR; 2.05), Cameroon (CM; 2.05), and Argentina (AR; 2.05) showed correspondingly low latent intercepts. Countries nearer the overall average, e.g., France (FR; 2.20), Germany (DE; 2.17), Italy (IT; 2.18), Switzerland (CH; 2.21), Poland (PL; 2.22), Nigeria (NG; 2.23), clustered around the middle of the latent distributions as well. All observed scores were below the burnout risk category cut-off 2.96^28^.

Even though some shifts in country positions between the two alignment methods can be expected, especially for the Netherlands which served as the fixed reference group, these shifts do not affect the overall pattern of results, which remained highly consistent across methods. Most countries retained their relative positions (|Δrank| ≤ 1) between the two alignment methods. A handful shifted more substantially when moving from the fixed (Netherlands-anchored) to the free solution. For instance, Italy (Fixed #7 → Free #14) and France (Fixed #9 → Free #15) dropped several ranks. Furthermore, smaller movements were observed for Poland, Japan, Chile, and Brazil. These shifts occurred in the mid-range where country means were more tightly clustered. Moreover, only a few countries showed changes greater than ± 2 ranks.

## Discussion

This study shows evidence of the validity of the BAT to model comparable burnout scores across 29 countries by means of measurement invariance alignment. Although both the fixed and free alignment methods yielded very similar patterns of country rankings in our data, the free alignment solution produced a warning indicating potential estimation problems. In contrast, the fixed alignment solution (anchored in the Netherlands) produced a stable distribution of intercepts. For this reason, we consider the fixed alignment results to provide the most reliable. The alignment analysis demonstrated that 92.62% of model parameters were invariant, with particularly strong invariance for the general burnout factor (95.69% of loadings) and all specific factors exceeding rule of thumb expectations. Furthermore, the McDonald’s omega coefficients were also highly acceptable across all samples. These findings provide evidence for validity and reliability that the BAT-12 can measure burnout complaints equivalently across diverse cultural contexts, supporting its use in multinational research and practice. The high level of measurement invariance observed (well above the rule of thumb thresholds) indicates that differences in BAT-12 scores between countries likely reflect differences in burnout experiences rather than measurement artifacts or cultural bias in item interpretation. These results align with the BESEM approach in previous countries without using the alignment approach but the multigroup approach across nine countries^12^.

While the measurement invariance alignment method supports the comparison of means between the countries, it must be noted that our samples were obtained through sourcing platforms and despite efforts to obtain demographic balance, the samples are not representative of the nations. However, as measurement alignment is specifically designed to be able to compare means across countries we provide these means for illustrative purposes.

Although country-level differences were observed in the sample-based latent means, the present study was not designed to explain those differences. The analyses did not incorporate contextual covariates such as industry composition, working hours, or broader economic conditions, and the country samples were not nationally representative. Accordingly, we avoid strong regional or cultural interpretation of these differences. The primary contribution of the study is psychometric: demonstrating that the BAT-12 can be modeled with a high degree of approximate invariance across the included country samples. The differences in country-level burnout scores should therefore be interpreted with caution given potential sampling biases: For example, when considering the observed scores, Saudi Arabia, Singapore, and Egypt showed the highest averages, while Colombia, Turkey, and Mexico showed the lowest. In the case of Saudi Arabia, systemic and cultural factors have been suggested as playing a significant role in shaping workplace dynamics and contributing to burnout^29^. Singapore has been described as a high-pressure, competitive environment that may contribute to extreme stress levels despite the nation’s economic affluence and efficiency^30^. Egypt’s high ranking highlights challenges that may be faced in African contexts, where socioeconomic and workplace stressors may combine to elevate burnout risks. New Zealand also appeared among the higher-scoring countries^31^, which is somewhat surprising given its geographical and cultural similarities to Australia, which was ranked lower in this study. However, all these patterns may primarily reflect the selection processes and demographic characteristics of participants recruited through crowdsourcing platforms in each country rather than true population-level differences.

This study’s primary contribution is in providing evidence for cross-cultural measurement invariance for the BAT-12, providing the psychometric foundation necessary to support its use for valid cross-national burnout research. While illustrative country comparisons are also presented, their interpretation must be tempered by considering the representativeness of the samples.

### Limitations and recommendations for future research

The multinational nature of our study is considered a key strength. However, a limitation is the representativeness of our sample. While efforts were made to match country-based quotas as closely as possible for gender, age, education, industry, employee position (managers vs. non-managers), and employer size—many of these quotas were not feasible to obtain in practice via our crowdsourcing platform. As such, future research could explore tighter adhesion to quotas to random sampling or consider demographic group specific sampling to provide further evidence of validity for the BAT-12. Furthermore, some lingering effects of the pandemic might also be present in some countries. Therefore, the burnout levels/rankings should be considered with some caution and subject to change pending even more robust sampling. Secondly, as the data are cross-sectional in nature we cannot comment on the stability of these burnout scores over time, or how other factors might influence the burnout levels over time. However, research has shown that aspects of burnout’s variance remain relatively stable over time^32^. Lastly, although the attention checks were intended to identify inattentive responding, we acknowledge that in a multinational panel design, failure on instructed-response items may also reflect cross-country differences in instruction processing, survey-taking behavior, or use of response anchors. Accordingly, these exclusions may not have been entirely neutral with respect to cross-country comparability.

### Practical implications

This paper has shown evidence for the validity that the BAT-12, the shorter version of the BAT-23, can be used across 29 countries by practitioners including occupational health practitioners, wellbeing and health officers, HR professionals and even team leaders. The tool offers opportunities for developing insight on an organizational level to the levels of burnout symptoms in the organization which can inform employee wellbeing strategy, intervention planning and impact tracking. This tool can also be leveraged by team leaders for check-ins (provided this happens in a safe environment and where managers are trained to do so) with their teams about healthy work-norms and ways of working. Team leaders can be trained how to interpret this instrument and how to observe when team members suffer from burnout symptoms. When team leaders are professionally trained they know how to respond and for example signpost an individual team member to the right help and support. Simultaneously team leaders can use these insights to act preventatively and collaboratively with the team ensure a right balance of work demands (for example creating clarity about the different roles in the team, helping solve conflicting tasks or streamlining work, supporting sufficient recovery time and helping the team to manage work pressure and workload) on one side and helping strengthen resources (for example autonomy and flexibility in the job, an inclusive environment, a sense of meaning at work and growth and learning opportunities) on the other side. Individual employees could also learn about symptoms of burnout and use these tools for self-reflection, helping them to learn how to set boundaries and respond effectively when they need either space for recovery or help for reducing work pressure and on the other side how they can enable themselves to better take care of their health holistically, understanding signals of health and intervene timely. With this instrument being validated to be consistent across 29 countries, multinational organizations can also leverage this tool internally to get insight across these countries and plan solutions cross culturally.

## Conclusion

This study provides evidence that the BAT-12 can be modeled with a high degree of approximate measurement invariance across 29 country samples. Overall, the findings support the cross-national comparability of the BAT-12 and strengthen its psychometric basis as a brief burnout assessment instrument. However, country-level mean differences should be interpreted cautiously given the study’s sampling limitations. The main contribution of the study is therefore psychometric: providing further support for the BAT-12 as a tool for cross-national burnout assessment in research and practice.

## Data Availability

The data and analyses for this study can be requested from the corresponding author upon reasonable request.
